# Enhancement of Wound Healing in Normal and Diabetic Mice by Topical Application of Amorphous Polyphosphate. Superior Effect of a Host–Guest Composite Material Composed of Collagen (Host) and Polyphosphate (Guest)

**DOI:** 10.3390/polym9070300

**Published:** 2017-07-22

**Authors:** Werner E. G. Müller, Dinko Relkovic, Maximilian Ackermann, Shunfeng Wang, Meik Neufurth, Andrea Paravic Radicevic, Hiroshi Ushijima, Heinz C. Schröder, Xiaohong Wang

**Affiliations:** 1 ERC Advanced Investigator Grant Research Group at the Institute for Physiological Chemistry, University Medical Center of the Johannes Gutenberg University, Mainz, Duesbergweg 6, 55128 Mainz, Germany; Shunwang@uni-mainz.de (S.W.); mneufurt@uni-mainz.de (M.N.); hschroed@uni-mainz.de (H.C.S.); 2 Fidelta Ltd., Prilaz baruna Filipovića 29, 10000 Zagreb, Croatia; Dinko.Relkovic@glpg.com (D.R.); andrea.paravicradicevic@glpg.com (A.P.R.); 3 Institute of Functional and Clinical Anatomy, University Medical Center of the Johannes Gutenberg University, Johann Joachim Becher Weg 13, D-55099 Mainz, Germany; maximilian.ackermann@uni-mainz.de; 4 Division of Microbiology, Department of Pathology and Microbiology, Nihon University School of Medicine, 30-1 Oyaguchi Kamicho, Itabashi-ku, Tokyo 173-8610, Japan; ushijima-hiroshi@jcom.home.ne.jp

**Keywords:** polyphosphate, microparticles, delayed wound healing, collagen, PAI-1, re-epithelialization, diabetic mice

## Abstract

The effect of polyphosphate (polyP) microparticles on wound healing was tested both in vitro and in a mice model in vivo. Two approaches were used: pure salts of polyphosphate, fabricated as amorphous microparticles (MPs, consisting of calcium and magnesium salts of polyP, “Ca–polyp-MPs” and “Mg–polyp-MPs”), and host–guest composite particles, prepared from amorphous collagen (host) and polyphosphate (guest), termed “col/polyp-MPs”. Animal experiments with polyP on healing of excisional wounds were performed using both normal mice and diabetic mice. After a healing period of 7 days “Ca–polyp-MP” significantly improved re-epithelialization in normal mice from 31% (control) to 72% (polyP microparticle-treated). Importantly, in diabetic mice, particularly the host–guest particles “col/polyp-MP”, increased the rate of re-epithelialization to ≈40% (control, 23%). In addition, those particles increased the expression of *COL-I* and *COL-III* as well as the expression the α-smooth muscle actin and the plasminogen activator inhibitor-1. We propose that “Ca–polyp-MPs”, and particularly the host–guest “col/polyp-MPs” are useful for topical treatment of wounds.

## 1. Introduction

Acute and, in particular, chronic wounds are a global health problem, with over 10 million people affected and ≈300,000 people hospitalized every year alone in the United States [1]. Non-healing wounds affect 3–6 million people in the United States; persons aged over 65 years represent 85% of these patients, with costs representing more than $3 billion per year [2]. Wound healing has been staged into the following phases; coagulation/inflammation, formation of granulation tissue, production of new structures and tissue, and finally, remodeling [3]. These complex processes are regulated by cytokines and growth factors and are decisively modulated by systemic conditions, e.g., diabetes. During the coagulation/inflammatory phase, blood platelets adhere to damaged blood vessels and initiate a release reaction resulting in the initiation of the blood-clotting cascade [1]. Blood platelets release an array of growth factors and cytokines as well as survival or apoptosis-inducing agents. Major factors involved are the platelet-derived growth factor and the transforming growth factors A1 and A2. In turn, inflammatory cells, including macrophages and leukocytes, are attracted, and release antimicrobial reactive oxygen species and proteases which remove non-self-bacteria and cell debris. Finally, the inflammatory phase is terminated by apoptosis during which inflammatory cells are eliminated. In the following proliferative/granulation phase, tissue repair starts a process that is controlled by growth factors produced by invading epidermal and dermal cells that via autocrine, paracrine, and juxtacrine pathways induce and maintain cellular proliferation and cellular migration [4]. The formation of granulation tissue allows epithelialization within the wound. During the final matrix remodeling and scar formation phase normal blood supply to the regenerated tissue is completed providing a suitable microenvironment for epidermal and dermal cell proliferation and migration, contributing to wound re-epithelialization and restoration.

**Figure 1:**
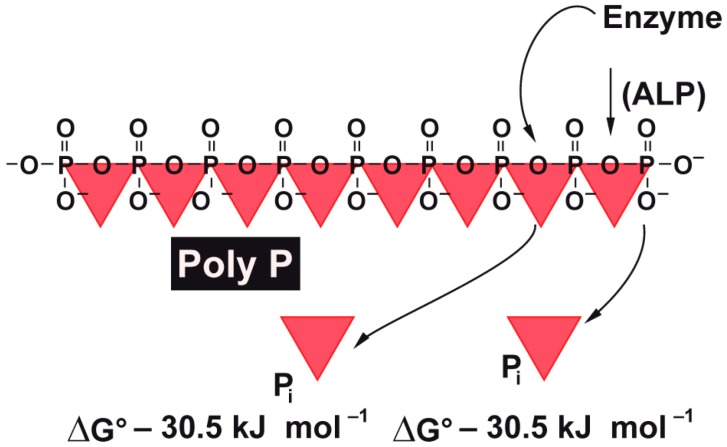
The linear inorganic polyphosphate (poly P) is composed of many phosphate (Pi) residues which are linked by high-energy phosphoanhydride bonds. If those “high-energy” phosphate bonds are cleaved, e.g., by enzymes (like the alkaline phosphatase), the “stored” energy is released (Δ*G*—30.5 kJ·mol^−1^).

The supply of metabolic energy to the regeneration zone of the wound is a critical parameter that influences wound-healing kinetics. This becomes overt on studying chronic wound metabolism. As an example, venous ulcers are attributed as a consequence of venous valvular insufficiency both in the deep and the superficial veins. This impairment results in blood backflow followed by an increased venous pressure [5] and increased blood vessel wall permeabilities. Similarly, complications during aging and diabetes show serious consequences, with both arterial and venous insufficiencies. In diabetic patients (diabetic ulcers) vascular impairment and cellular deficiencies are frequently parallel to microvascular pathologies [6]. The biochemical consequences of impaired vascularization are reduced cell metabolism and supply of oxygen required for energy production by means of ATP [7]. It is well thematized and proven that intracellular ATP levels are directly correlated with the efficiency of wound healing and tissue regeneration. However, until recently it remained obscure as to which metabolic energy storages exist in the extracellular space of the tissue. Now it has become more and more obvious that polyphosphate (polyP), comprising up to several hundreds of phosphate units linked with “high-energy” phosphoanhydride bonds, contributes as a “metabolic fuel” [8,9] to the establishment and maintenance of the extracellular structural and functional organization. ATP is present (if at all) solely in minute concentrations in the extracellular space [10] where it acts as a signaling molecule only [11]. In this place ATP is prone to hydrolytic cleavage through the alkaline phosphatase (ALP) [12]. It is exactly this enzyme that also hydrolyzes polyP [13]. Consequently—as in any biochemical cleavage reaction of energy-rich phosphoanhydride bonds—enzymatic hydrolysis of polyP by the ALP will release metabolically useful chemical energy [14] as well as some heat (see scheme in Figure 1). It has been shown that metabolic chemical energy, in the form of ATP, is required for endothelial cells to grow and differentiate—especially during the maintenance and regulation of angiogenesis [15]. For the preparation of Ca^2+^–polyP particles we introduced a new technology; the amorphous microparticles were obtained by co-precipitation of the highly soluble Na^+^–polyP in the presence of a large surplus of CaCl_2_ [8]. Very recently we also succeeded in preparing a collagen (host) and Ca–polyP (guest) hybrid material which displayed pronounced potencies for the induction of cell proliferation of primary human osteoblasts under in vitro conditions [16].

polyP is a remarkable inorganic macromolecule. It exists as a polymer of around 100 phosphate units in many cells and also in the blood, in especially high concentrations in blood platelets [17]. This physiological polymer has been implicated in the synthesis of amorphous calcium phosphate from amorphous calcium carbonate after its enzymatic hydrolysis to ortho-phosphate during bone formation [18]. In addition, polyP as a (potential) metabolic fuel acts as a suitable bio-ink in three-dimensional tissue printing [9]. The major polyP stores in mammals are the blood platelets which play a crucial role during all phases of wound healing, including coagulation, immune cell recruitment/inflammation, and angiogenesis as well as in remodeling. The ALP activity is highest during the phase of granulation/tissue formation [19]. Hence, there was an obvious need to clarify the effect of polyP, incorporated as a topical wound dressing, on wound-healing kinetics. However, a formulation for polyP needed to be developed in which polyP is released in a slower rate, which prevents rapid enzymatic hydrolysis. Furthermore, those salts must be amorphous, in order to be biologically active. This task was achieved by the fabrication of this polymer as an amorphous Ca^2+^ complex in 100- to 300-nm large microparticles [20]. In this form polyP retains its propensity to undergo enzymatic hydrolysis and shows morphogenetic activity. 

**Figure 2:**
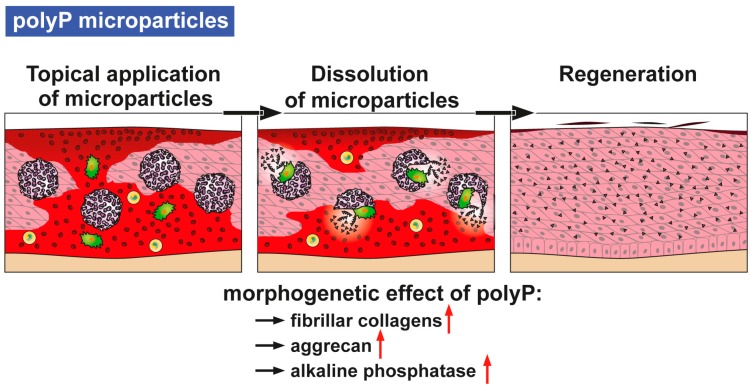
Proposed action mode of polyP, in the form of polyP microparticles, as a morphogenetically active and wound-healing-promoting physiological inorganic polymer. After topical application of the polyP microparticles (with collagen as host and polyP as guest) they undergo dissolution in the impaired tissue, as a result of ALP enzymatic hydrolysis. Then, polyP is known to activate the genes e.g., those encoding fibrillar collagens, aggrecan and the ALP. The experimental data given verify that these polyP microparticles accelerate wound healing and tissue regeneration.

Furthermore, fibrillar collagen remodeling during wound healing, especially during the transition from granulation tissue to scar tissue, is dependent on a continuous synthesis and catabolism of the respective collagen types [4]. In a previous contribution we could demonstrate that the expression of the fibrillar collagen genes, of types I, II, and III, are strongly upregulated in vitro by polyP microparticles, especially if they are enriched with retinol [21]. In addition, for the genes encoding for collagens, the steady-state-expression levels of aggrecan [22] (a major regulatory proteoglycan also during wound healing) and of ALP (an enzyme found in the first stage of acute wound healing) [23], increase in response to polyP [24]. These results prove the morphogenetic activity of polyP, as summarized [9] (Figure 2). Hence, it is the aim of the present study to test both in vitro and in vivo to evaluate if polyP in a suitable formulation(s) elicits a beneficial effect on experimental wound healing in mice. Three different forms of polyP microparticles (MPs) have been fabricated, polyP in the form of a Ca^2+^ salt (Ca–polyP-MPs) and a Mg^2+^ salt (Mg–polyp-MPs) as well as a hybrid material together with collagen (col/polyP-MPs). Collagen has been selected as additional component (as host) since this structural macromolecule has been proven to act as a scaffold for wound dressings and facilitates cell attachment, growth and differentiation [25]. The animal experiments were performed both with normal (C57BL/6) and diabetic mice. As a morphogenetic parameter for the effect of the polyP particles in vivo, the potency to induce gene expression of collagen has been chosen. More specifically, the expression of *collagen type I* (dominant in skin and vascular ligature) and the reticulate *type III* (reticular fibers) was determined; these types of collagen are critical for wound healing [26]. In addition, the steady-state-expression levels for *α-smooth muscle actin* (α-SMA) [27] and for *plasminogen activator inhibitor-1* (PAI-1) [28], both genes that are strongly upregulated during wound healing, were determined. Based on the data obtained it is concluded that both the “Ca–polyP-MPs” and the “col/polyP-MPs” positively accelerate the kinetics of wound healing and are (potentially) beneficial for the topical treatment of wounds in humans as well.

## 2. Materials and Methods

### 2.1. Materials

Na–polyphosphate (Na–polyP) with an average chain length of 40 phosphate units was from Chemische Fabrik Budenheim (Budenheim, Germany). The general formula of this Na–polyP is [NaPO_3_]*_n_*[NaPO_3_(OH)]_2_, where *n* is ≈40. Rat tail collagen type I was obtained from Shenzhen Lando Biomaterials Co., Ltd. (Shenzhen, China).

### 2.2. Amorphous PolyP Microparticles

Amorphous polyP particles were prepared from Na–polyP in the presence of a surplus of divalent ions (Ca^2+^ or Mg^2+^). The final stoichiometric ratio between these ions and the polyP polymer (with reference to one charged phosphate unit) is close to 2. The procedure is outlined in brief.

Mg–polyP particles were basically prepared as described [29]. In short, 3.86 g of MgCl_2_·6H_2_O (#A537.1, Roth, Karlsruhe, Germany) was dissolved in 25 mL of distilled water and added dropwise to 1 g of Na–polyP, likewise in 25 mL of distilled water. During particle formation the suspension was kept at pH 10 and then stirred for 12 h. The microparticles formed were collected by filtration, washed with ethanol and dried at 50 °C. The sample was termed “Mg–polyP-MP”. 

Ca-polyP particles were fabricated from 2.8 g of CaCl_2_·2H_2_O (#223506; Sigma-Aldrich, Taufkirchen, Germany) (25 mL of distilled water) and 1 g of Na–polyP (25 mL water) at room temperature [20]. The microparticles are formed in the pH 10 suspension during the 12-h stirring period. Then the microparticles were collected by filtration, washed with ethanol and dried at 50 °C. They are termed “Ca–polyP-MP”. 

Collagen-polyP host–guest hybrid particles were prepared as follows: A suspension of 20 mL of collagen (containing 0.2 g of fibrous collagen material) was added to 50 mL of aqueous Na–polyP solution (containing 0.2 g of solid salt). The developing suspension was kept at pH 9 (with NaOH) and stirred for 4 h at room temperature. The resulting suspension was filled into a syringe (aperture of 2 mm), clamped into an automatic pump, and injected with a speed of 1 mL/min into a CaCl_2_ bath (3 g of CaCl_2_·2H_2_O per 100 mL) composed of ethanol:acetone (1:2 *v*/*v*). Ethanol and acetone prevented shrinkage of the particles, reduced the surface tension and extracted water. Under those conditions the organic solvents are considered not to alter the biological properties of collagen [30]. The suspension was stirred overnight. The particles were collected by filtration and washed twice in acetone. Then the particles, termed “col/polyP-MP” (host–guest particles), were dried at room temperature. A schematic outline of this procedure, and preparation of the host (collagen)–guest (Ca–polyP microparticles) hybrid material, has been given previously [16].

Prior to use all particles were sieved through a 500-µm mesh net. 

### 2.3. Microstructure Analyses

Scanning electron microscopic (SEM) imaging was performed using a Hitachi SU-8000 electron microscope (Hitachi High-Technologies Europe GmbH, Krefeld, Germany). Reflection electron microscope (REM) was performed in a Philips XL30 microscope (Philips, Eindhoven, The Netherlands) at 15 KeV and 21 µA. The samples were coated with 20–25 Å gold in an argon atmosphere.

For energy-dispersive X-ray (EDX) spectroscopy an EDAX Genesis EDX System attached to the scanning electron microscope (Nova 600 Nanolab, FEI, Eindhoven, The Netherlands) was used, using the operation mode 10 kV and a collection time of 30–45 s.

### 2.4. Cultivation of MC3T3-E1 Cells

Mouse calvaria cells MC3T3-E1 (ATCC-CRL-2593, #99072810) were cultivated in α-MEM medium (Gibco/Invitrogen, Darmstadt, Germany) enriched with 20% fetal calf serum (FCS, Gibco, Schwerte, Germany). In addition, the medium contained 2 mM l-glutamine, 1 mM Na-pyruvate and 50 µg/mL of gentamicin. For the gene expression studies the cells were cultivated in 24-well plates (Greiner Bio-One, Frickenhausen, Germany). The cells were seeded at a density of 5 × 10^3^ cells/well. After an incubation period of 4 days the cells were harvested and subjected to PCR analysis. 

Na–polyP was added to the culture/serum, after stoichiometric complexation with Ca^2+^ (molar ratio of 2:1/phosphate monomer: Ca^2+^). Prior to addition of the microparticles to the culture they were washed twice in medium for 3 min. 

### 2.5. Animals

Genetically diabetic male mice, BKS.Cg-m + Lepr^db^/+Lepr^db^ (db/db), aged 6 and 7 weeks at arrival (Charles River, Calco, Italy), and a common inbred strain of laboratory mouse, male C57BL/6, aged 7 weeks at arrival were used. The diabetic mice were markedly hyperglycemic (mean blood glucose: 527 ± 9 mg/dL), compared to non-diabetic animals (205 ± 9 mg/dL) with all details given previously [31]. For acclimatization, a minimum of 5 consecutive days prior to the experiments in the laboratory animal house was chosen. All animals received a detailed physical examination from the resident veterinarian to confirm that the animals were in a good, adequate state of health. Daily observations were performed at the time of delivery of the animals, during the total period of acclimatization and also throughout the duration of the study.

Housing of the animals: Mice were kept in solid bottomed cages (polysulfone type III H; Tecniplast, Buguggiate, Italy) with dimensions of 425 mm × 266 mm × 185 mm. The animals were kept on 3–4 cm thick ALPHA-dri dust free bedding (pure cellulose fiber, uniform particle size 5 mm sq, highly absorbent; LBS Serving (RH6 0UW, Horley, UK)). Each cage was provided with a nestlet and Des Res Standard (for mice) 16 cm long × 12 cm wide × 8 cm high; LBS Serving. The animals were maintained under standard laboratory conditions (temperature 22 ± 2 °C, relative humidity (55 ± 10)%, 15–20 air changes per h, 12 h artificial lighting/12 h darkness per day (7.00 a.m. lights on–7.00 p.m. lights off)). The animals had free access to food VRF1 (P) (Akronom KfT, Budapest, Hungary) and water distributed in bottles (Tecniplast) and filled with drinking water from the municipal water supply.

### 2.6. Permissions

All animal-related research was conducted in accordance with 2010/63/EU and national legislation regulating the use of laboratory animals in scientific research and for other purposes (Official Gazette 55/13). An Institutional Committee on Animal Research Ethics (CARE-Zg) had overseen that animal-related procedures did not compromise animal welfare. All experiments conducted in studies described herein were performed under the institutional ethics committee approval number CAREZG_13-06-14_49 EP/2016 (SP-167-15 and SP-167-16). The approval number from the Ministry of Agriculture, Republic of Croatia was KLASA: UP/I-322-01/15-01/108, URBROJ: 525-10/0255-16-8.

### 2.7. Experimental Procedures in Wound-Healing Studies

In each study, mice were divided into groups (six animals per group). The interscapular region was shaved, and depilatory cream Veet (Slough, UK) was applied onto the shaved region and removed 2 min after application on day 2. The interscapular region was disinfected and, utilizing strictly aseptic procedures, a single full-thickness excisional wound 8-mm in diameter was inserted midline with a sterile, disposable biopsy punch, thus exposing the underlying fascia muscularis as described. 

The test samples were applied in the powder form (100%) and directly spread into the wound beds immediately post-wounding at day 0. The wound was covered by Tegaderm Wound dressing (3M, St. Paul, MN, USA) which remained there until the end of the study.

Postoperative pain control included daily s.c. injections of 4 mg/kg carprofen (5% Norocarp (Pfizer, New York, NY, USA)–50 mg/mL; a 1:20 dilution was made in sterile, deionised water; 30 µL were injected into an animal) for two days post-wounding at day 0 until day 2. Skin samples were collected at time points specified in the study. Prior to skin sampling all animals were humanely killed with an overdose of ketamine (Taj Pharmaceuticals, Newcastle, UK)/xylasine (KHBoddin, Hamburg, Germany) administered via the intraperitoneal route. 

### 2.8. Sample Analysis: Wound Excision and Morphometry

After terminating the experiments, the wound areas and the surrounding healthy tissues were excised post mortem (1 cm × 2.5 cm, rectangular shape), clamped onto a strip of paper and stored in 10% formalin for histological assessment.

Morphometry: Slides cut from paraffin blocks were stained with hematoxylin–eosin [32]. Morphometric evaluation of the degree of re-epithelialization was performed using a Zeiss Axioskop 2 Plus microscope and an Axiovision program (Zeiss, Oberkochen; Germany). The magnification used was 100×. Re-epithelialization is expressed as length of newly formed epithelium (given in mm) and percentage of the wound diameter covered with a new epithelial layer. 

### 2.9. Gene Expression Studies

Animals: The technique of quantitative real-time reverse transcription polymerase chain reaction (qRT-PCR) was applied to determine semi-quantitatively the effect of the polyP particles on wound healing. Formalin-fixed paraffin-embedded (FFPE) tissue samples were used and RNA was extracted from those [33]. The reactions were performed in 1.5 mL Eppendorf tubes and all incubation steps were performed in a thermal cycler (Thermomixer comfort, Eppendorf, Hamburg, Germany). Random primers were used in concentrations of 250 ng/reaction. RNA was mixed with primers together with 1 µL of 10 mM dNTPS, incubated for 5 min at 65 °C and then cooled on ice for 1 min. To each sample the following components were added to the reaction: 4 µL 5 × buffer (Thermo Fisher Scientific, Langenselbold, Germany), 1 µL 0.1 M dithiothreitol (DTT), 1 µL RNaseOUT (RNAse inhibitor, Thermo Fisher Scientific, Schwerte, Germany) and 1 µL Superscript III reverse transcriptase (Thermo Fisher Scientific). The mixture was incubated as follows: 5 min at 25 °C, 60 min at 50 °C and 15 min at 70 °C. When cooled down the mixture was diluted 5 times with RNase free water and used for qPCR. The following primer pairs for mouse genes were used: for *procollagen type I*α (COL-I; AK075707) Fwd: 5′-AGGCTGACACGAACTGAGGT-3′ and Rev: 5′-ATGCACATCAATGTGGAGGA-3′; *collagen type III* α*I* (COL-III; P08121) Fwd: 5′-GCTGTTTCAACCACCCAATACAGG-3′ and Rev: 5′-CTGGTGAATGAGTATGACCGTTGC-3′; α-*smooth muscle actin* (α-SMA; NC_000074.6) Fwd: 5′-CAGGGAGTAATGGTTGGAAT-3′ and Rev: 5′-TCTCAAACATAATCTGGGTCA-3′; *plasminogen activator inhibitor-1* (PAI-1; NC_000071.6) Fwd: 5′-CTGCAGATGACCACAGCGGG-3′ and Rev: 5′-AGCTGGCGGAGGGCATGA-3′. The *hypoxanthine phosphoribosyltransferase 1* gene was used as house-keeping gene (HPRT1; NM_013556.2) Fwd: 5′-TGGATACAGGCCAGACTTTG-3′ and Rev: 5′-GTACTCATTATAGTCAAGGGCATAT-3′. All reactions were carried out at least in duplicate and the results were analyzed by a 2^-∆∆*CT*^ method [33]. 

MC3T3-E1 cells: The cells were harvested and RNA was extracted. Then the RNA was subjected to qRT-PCR analysis for *collagen type I,* α *1* (COL-I, NM_007742) using the primer pair Fwd: 5′-TACATCAGCCCGAACCCCAAG-3′ and Rev: 5′-GGTGGACATTAGGCGCAGGAAG-3′ and for *collagen type III,* α *1* (COL-III; NM_009930) the pair Fwd: 5′-GCTGTTTCAACCACCCAATACAGG-3′ and Rev: 5′-CTGGTGAATGAGTATGACCGTTGC-3′. In this series of experiments the expression of *glyceraldehyde 3-phosphate dehydrogenase* (GAPDH; NM_008084) was chosen as a reference gene; the primer pair Fwd: 5′-TCACGGCAAATTCAACGGCAC-3′ and Rev: 5′-AGACTCCACGACATACTCAGCAC-3′ was chosen. The determinations were performed in an iCycler (Bio-Rad, Hercules, CA, USA) with the respective iCycler software.

### 2.10. Statistical Analyses

The gene expression studies for the incubation experiments in vitro as well as for the re-epithelialization determinations in vivo are expressed as mean (± standard error of mean; independent two-sample Student’s *t*-test; Mann–Whitney U-test) [34]. The PCR studies forming the in vivo experiments are given as Box plot analyses [35]. 

## 3. Results 

### 3.1. Amorphous PolyP Microparticles

**Figure 3:**
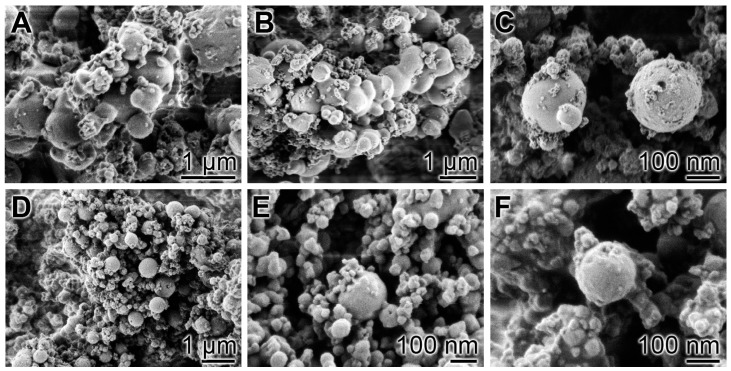
Morphology of “Ca–polyP-MPs” and “Mg–polyP-MPs”; SEM analysis. (**A**–**C**) “Ca–polyP-MP” and (**D**–**F**) “Mg–polyP-MP”.

For both the in vivo and the in vitro tests Na–polyP powder and the microparticles “Mg–polyP-MP”, “Ca–polyP-MP” and “col/polyP-MP” were prepared. The size of the globular “Ca–polyP-MP” particles varied between 100 nm and 800 nm with an average of 450 ± 170 nm (*n* = 60) (Figure 3A–C). The surface of the particles showed pores of sizes around 10 nm only at a higher magnification. 

A similar morphology is characteristic for the likewise globular “Mg–polyP-MP” microparticles (Figure 3D–F). These particles are more homogeneous than the “Ca–polyP-MP” with an average of 170 ± 65 nm. Broken particles revealed that the particles are close to compact. 

**Figure 4:**
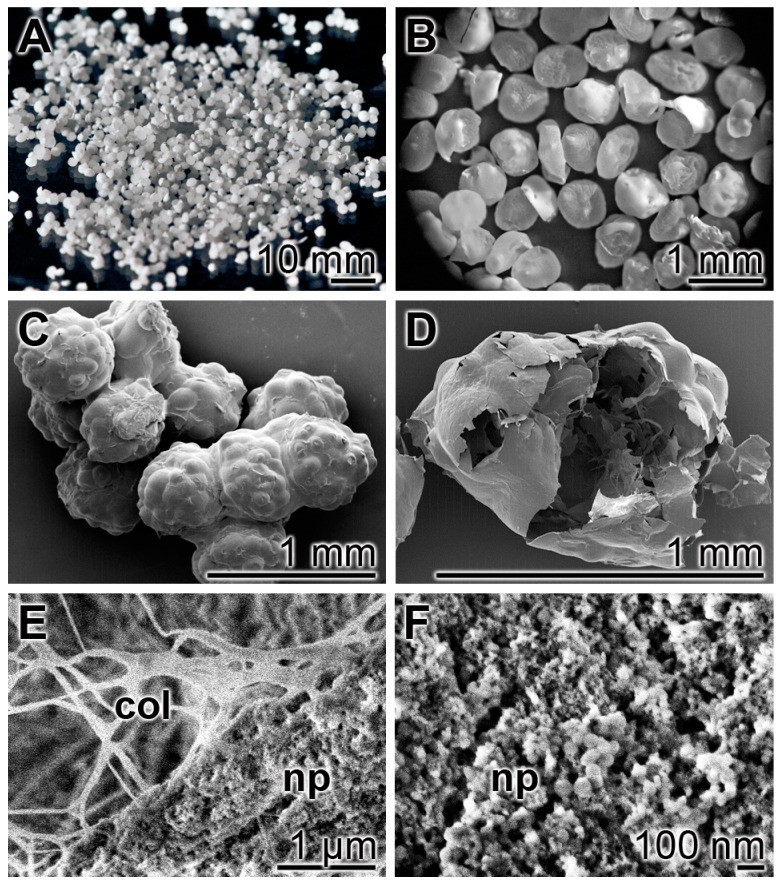
Electron microscopic images of the host–guest “col/polyP-MPs”; (**A**) to (**D**): Reflection electron microscope (REM) and (**E**) and (**F**): SEM. (**A**) and (**B**) At a lower magnification (REM) the particles appear as globular to disc-like particles. (**C**) The intact particles have on their surfaces ball-like protrusions which (**D**) proved to be gas bubbles in broken particles. (**E**) and (**F**) At SEM magnification it can be identified that the scaffold material is built of a collagen (col) fiber framework around which nanoparticles (NPs), within the microparticles, are arranged. (**F**) At the higher magnification it can be resolved that the polyP nanoparticles, formed at the collagen scaffold, have a homogeneous morphology with a diameter of ≈ 30 nm.

The “col/polyP-MP” are less spherical than the other two particles. The particle size distribution is fairly homogeneous with an average diameter of 450 ± 0.190 µm (Figure 4A,B). The morphology varies from almost globular to close to disc-like. At REM magnification it is already seen that the intact particles show ball-like protrusions (Figure 4C) that proved to be gas bubbles (Figure 4D). At the higher SEM magnification it is revealed that the solid material that surrounds the gas vesicles is built of a collagen scaffold around which the polyP particles are arranged (Figure 4E). At a higher magnification the size of the polyP nanoparticles can be determined with ≈30–50 nm (Figure 4F). In those images it can be seen that basically the distribution of the collagen scaffold within the particles is homogeneous (Figure 4). The Ca–polyP particles become associated with the collagen fibers during the precipitation with CaCl_2_. The distribution of those particles onto the surface of the fibers is dense (Figure 4E,F). 

**Figure 5:**
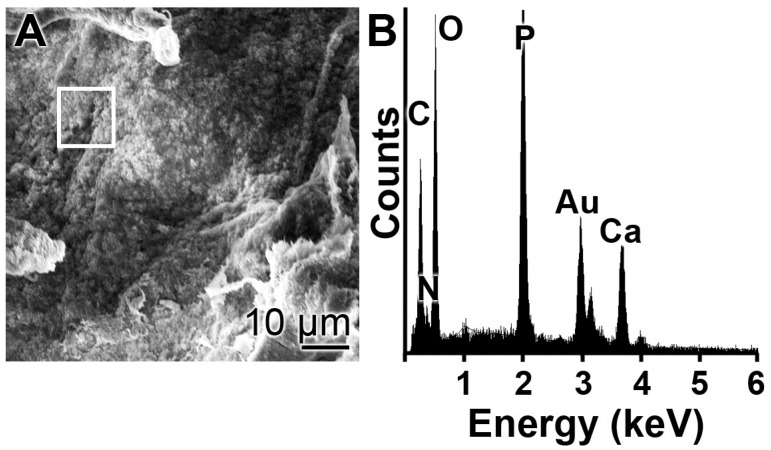
Energy-dispersive X-ray (EDX) analysis of “col/polyP-MP”. (**A**) SEM analysis and (**B**) EDX spectrum. The prominent element peaks (C, N, O, P and Ca) are marked. The Au peak originates from the gold surface after sputtering. The white square in (**A**) delimits the area selected for the EDX analysis.

For the analysis of the elements, present in the “col/polyP-MP” particles, EDX spectroscopy was applied. As documented in Figure 5, the spectrum shows the characteristic signals for C, N, O, P and Ca. The C, N O signal peaks can be attributed to the collagen framework and the P and Ca peaks to Ca–polyP. In previous studies it is reported that both the “Ca–polyP-MP” [20] and the “Mg–polyP-MP” particles are amorphous [28]. It has also been verified that the “col/polyP-MP” have this state (data not shown). 

### 3.2. Potency of polyP to Change the Steady-State Expression of Collagen Genes in MC3T3-E1 Cells In Vitro

**Figure 6:**
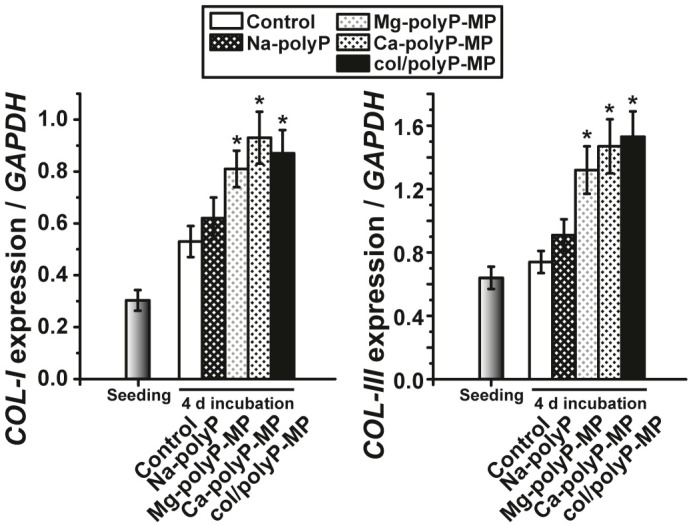
Gene expression levels of the two collagen types, *collagen type I* (COL-I), and *collagen type III* (COL-III) in MC3T3-E1 cells exposed to 50 µg/mL of “Na–polyP”, “Mg–polyP-MPs”, “Ca–polyP-MPs” or “col/polyP-MPs”; the controls received no additional component. After the 4-day incubation period the cells were harvested, their RNA was extracted and the steady-state levels of collagen expression were determined by qRT-PCR. The expression levels at time 0 (seeding) and time 4 days are correlated with the one of the housekeeping genes *GAPDH*. * *p* < 0.01 (*n* = 6 parallel experiments; Student’s *t*-test) with respect to the values of the controls.

Mouse calvaria MC3T3-E1 cells were cultivated in medium/serum as described under “Materials and Methods”; the seeding cell concentration was 5 × 10^3^ cells/well [24-well plates]. The cultures received either no additional component (controls) or were exposed to 50 µg/mL of “Na–polyP”, “Mg–polyP-MPs”, “Ca–polyP-MPs” or “col/polyP-MPs” and incubated for 4 days. Then, the cells were harvested, and RNA was extracted which was subjected to PCR analysis to assess the expression levels for *collagen type I* and *collagen type III*. The data are summarized in Figure 6. It is striking that, under the conditions used, “Na–polyP” did not change the expression levels for either collagen type. However, a significant upregulation of the steady-state-expressions to about 2-fold was measured for *collagen type I* and also for *collagen type III* in the experiments with “Mg–polyP-MPs”, “Ca–polyP-MPs” and “col/polyP-MPs”. 

### 3.3. Effect of polyP Application on Re-epithelialization in C57BL/6 and db/db Mice

**Figure 7:**
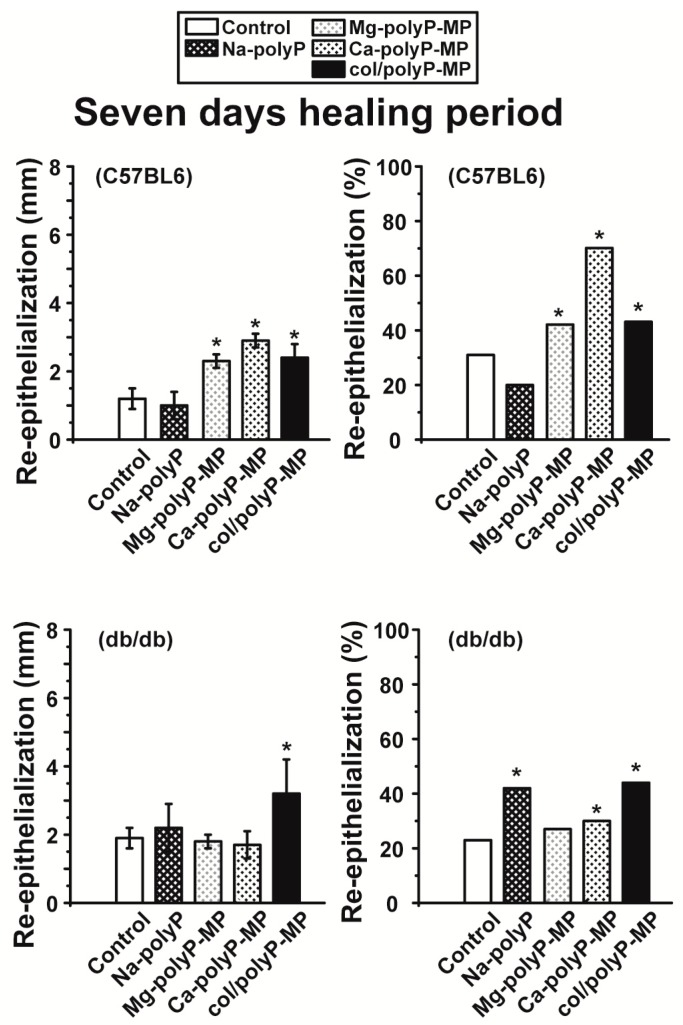
Effects of different polyP formulations on wound-healing kinetics (7 days after setting the wound), measured on the basis of re-epithelialization (in percent) which is calculated by dividing the degree of re-epithelialization (in mm) by the wound diameter (mm) × 100. For the study both wild-type (C57BL/6) and db/db mice were included (experiment #SP-013-16). The values for both the controls (without polyP) and the different polyP formulations, “Na–polyP”, “Mg–polyP-MP”, “Ca–polyP-MP” and “col/polyP-MP”, are given. The data are presented as mean ± SEM. The significance is * *p* < 0.05; *n* = 6 animals (Student’s *t*-test) [* *p* < 0.05 versus negative control according to the Mann–Whitney U-test].

**Figure 8:**
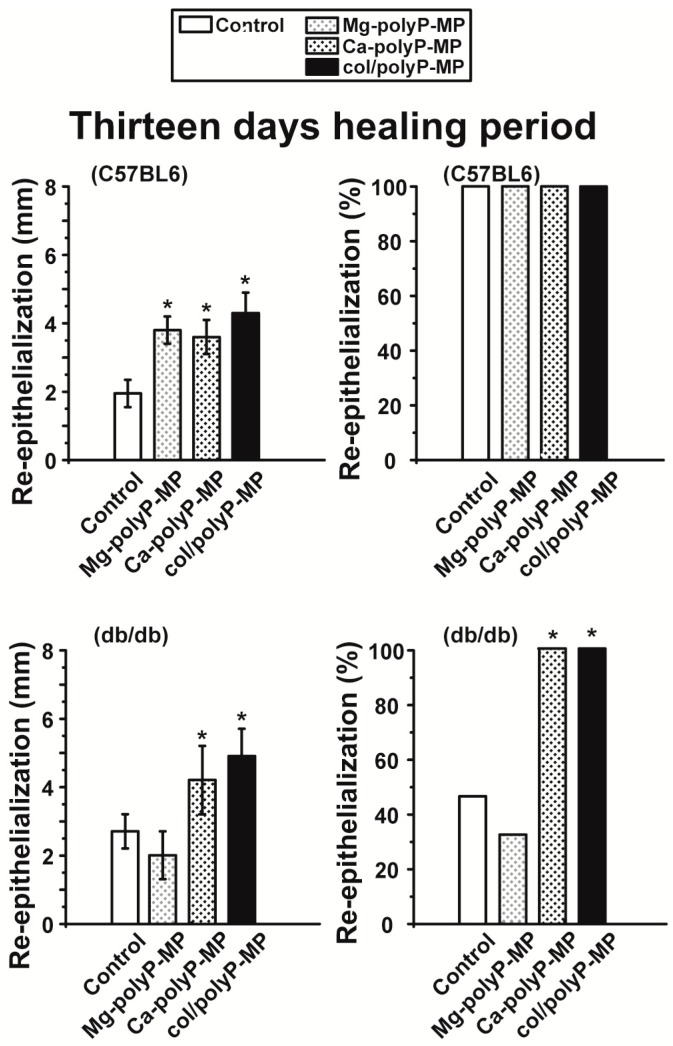
The effects of the tested materials on wound re-epithelialization in C57BL/6 and db/db mice on day 13. The data are presented as mean ± SEM.

Groups of six specimens each of C57BL/6 and db/db mice were used for the study. Each mouse received one defined wound with a diameter of 8 mm. The wounds were supplied once with 3 mg of the respective samples immediately after setting the wound. After a healing period of 7 days (Figure 7) or 13 days (Figure 8) the experimental animals were analyzed for the degree of re-epithelialization. 

In the present study a delayed wound healing in diabetic (db/db) mice is described in comparison to normal mice. The results revealed that after a 7-day healing period (Figure 7) the C57BL/6 mice recovered with a 31% score of re-epithelialization (controls), while the db/db mice showed only 23% re-epithelialization. At day 7 some polyP formulations already showed a distinct beneficial effect on the wound healing kinetics. Statistical significant is the effect of “Ca–polyP-MP” in C57BL/6 mice with a score of re-epithelialization of 72%; somewhat lower is the positive wound healing effect of “Mg–polyP-MP” (40%) and “col/polyP-MP” (44%). In contrast, “Na–polyP” proved to be ineffective. Very distinctive is also the beneficial effect of polyP in db/db mice. In this model “Na–polyP” increased the rate of wound healing to 42%, if checked by the rate of re-epithelialization. Likewise significant is the effect of “Ca–polyP-MP” (30% re-epithelialization) and “col/polyP-MP” (44%) in diabetic mice. 

The wound healing velocity after the 13-day healing period is also very pronounced. While in the experiments with normal mice both the controls and the polyP-treated wounds had already re-epithelialized 100%, the diabetic mice (controls) showed only 48% re-epithelialization (Figure 8). In this series of experiments with both “Ca–polyP-MP” (score of 100%) and “col/polyP-MP” (100%) the epithelium totally covered the wound. 

It is worth mentioning that the Ca–polyP microparticles are more efficient under the in vivo conditions than Na–polyP or Mg–polyP, as demonstrated previously by showing that the Ca-polyP particles are less soluble compared to Na–polyP/Mg–polyP and hence are less susceptible to the ALP [20], an enzyme which is highly expressed during the granulation phase [19]. This implies also that the release kinetics of Ca–polyP from the particles is much slower compared to the polymer from Na–polyP or Mg–polyP and hence is longer effective.

### 3.4. Expression of Selected Genes in Tissue from the Regenerating Wound by qRT-PCR

**Figure 9:**
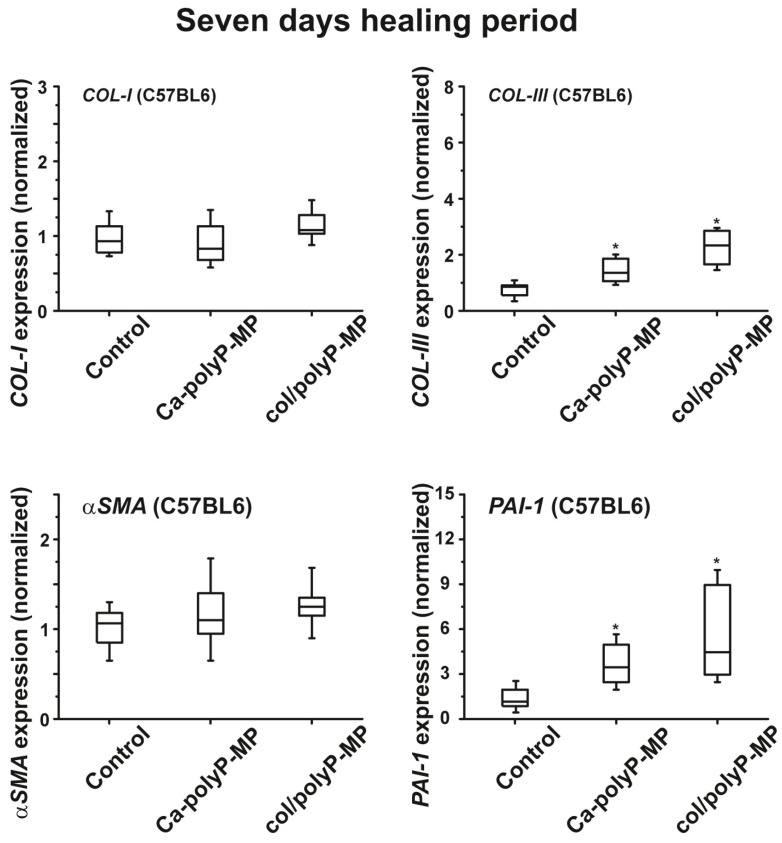
Upregulation of the collagen genes, *procollagen type Iα* (*COL-I*) and *collagen type III* α*I* (*COL-III*), in tissue from wild-type animals treated with the microparticles “Ca-polyP-MP” and “col/polyP-MP” (experiment #SP-013-15). The healing period was 7 days. The genes encoding *procollagen type I*α (COL-I) and *α-smooth muscle actin* (α-SMA) remained unchanged. The significance (Box plot characteristics) are given by comparing the values of controls (no polyP added) with the values measured for the “Ca–polyP-MP” and “col/polyP-MP” treated wounds. The horizontal bars within the boxes indicate the median value; * *p* < 0.005 (*n* = 6 animals).

RNA was extracted from FFPE-fixed tissue samples and then subjected to qRT-PCR. As a marker for cell migration and tissue re-organization the expression of the genes encoding for *procollagen type I*α (COL-I) and *collagen type III* α*I* (COL-III) have been chosen. As a measure for the granulation efficiency the genes α-*smooth muscle actin* (α-SMA) and *plasminogen activator inhibitor-1* (PAI-1) have been selected. The studies were performed with wild type mice. As summarized in Figure 9 it became evident that after the 7-day incubation period the transcript levels for the *collagen* marker gene *COL-III* and for *PAI-1* significantly upregulate in tissue taken from the wound area that has been treated with powder composed of polyP microparticles (“Ca–polyP-MP” and “col/polyP-MP”). With respect to the α-*SMA* gene or *COL-I* gene no significant change is seen. 

**Figure 10:**
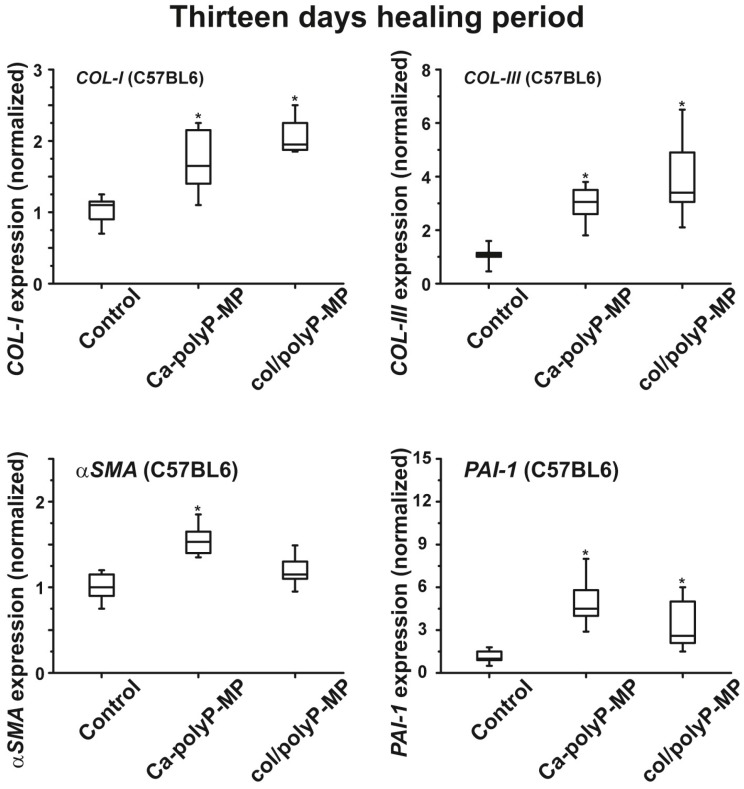
Changes of the steady-state expression of the genes *COL-I* and *COL-III* as well as of the genes α-*SMA* and *PAI-1* in wild-type animals after treatment with polyP microparticles. The healing period was 13 days. In all three series of experiments the expression levels of genes in tissue from regenerating wounds that were treated with polyP microparticles (“Ca–polyP-MP” and “col/polyP-MP”) significantly increased compared to control; * *p* < 0.005 (*n* = 6 animals).

After the longer healing period of 13 days the expression of all four marker genes for wound healing and tissue regeneration becomes upregulated by up to 1.6-fold (compared to the controls) for *COL-I*, and up to 4.6-fold for *COL-III*, 1.5-fold for α-*SMA* and 5.1-fold for *PAI-1* (Figure 10). 

## 4. Discussion 

It is very obvious that the therapy/care of both acute and chronic wounds has to begin with the understanding of the molecular and cellular etiology of the components present within each wound bed [1]. The failure in the venous and arterial blood flow and the balance of the levels of cytokines and growth facts in the wound area are a priority. In addition, systemic factors such as nutritional status, immunosuppression and infection contribute to the kinetics of wound healing. Also, the imbalance of the growth and differentiation state and rate of the cells involved, mainly fibroblasts, determine the functional quality of the hyperproliferative wound margin [2]. In addition, an imbalance of the pro-inflammatory cytokines and an uncontrolled enzymatic environment predisposes and causes delayed healing. Finally, local tissue hypoxia in concert with a repetitive ischemia–reperfusion injury accelerates pathogenesis in chronic wounds.

The first factors produced during the wound healing process originate from the blood platelets (platelet-derived growth factor) and the fibroblasts (fibroblast growth factor) (reviewed in [36]). One secret why blood platelets comprise a prime role during hemostasis is that they release coagulation factors that stop bleeding and cause clumping and clotting. However, as outlined in the “Introduction” they are filled with an inorganic polymer, polyP, that has recently been implicated in the extracellular energy supply [8]. In a very recent thorough review [37] the role of polyP as a signaling molecule in mammalian cells, causing an increased metabolism of mitochondria, has been highlighted. As examples, the signaling role of polyP between astroglial cells has been elucidated and the function as an amplifier proinflammatory response has been established [38]. Since the (potential) solution of the problem of the origin of polyP is the polymer delivering metabolic fuel to the cells we asked in the present study if the accelerating and anabolic effect of polyP on mineralization of bone cells can be applied also to cells, e.g., fibroblasts, controlling wound healing in vitro (fibroblasts) and in vivo (mouse wound healing model). Besides being a signaling molecule that migrates across the tiny spaces between adjacent cells, polyP has been proven to be a metabolic signal that regulates cell growth and differentiation, providing polyP with morphogenetically active properties. Examples are the induction of osteoinductive cytokine(s) and enzymes, like the ALP, whose dephosphorylation reactions result in an increased rate of diffusion of molecules into and inhibition of them to diffuse out the cells (scheme in Figure 2). 

The major studies, summarized here have been performed both with wild-type (C57BL/6) mice and diabetic (db/db) mice in order to bring together in a comparative manner results from the topical effects of polyP during acute and chronic wound healing. It is interesting to note that db/db mice have a genetic mutation in their leptin production pathway and/or leptin receptor signaling in the hypothalamus and, by that, lose control of satiation [39]. Mice having a homozygous mutation of the leptin gene show a phenotype of hyperphagia, extreme obesity, diabetes, neuroendocrine abnormalities, and infertility [40]. This exact gene has been found to be induced by polyP in mouse calvaria cells MC3T3-E1 cells in vitro [41]. We introduced for the study, summarized here, in addition to the already proven amorphous Ca–polyP particles (“Ca–polyP-MP”) [20] and the amorphous Mg–polyP microparticles (“Mg–polyP-MP”), a new hybrid polyP formulation with collagen as a scaffold, around which the Ca–polyP nanoparticles are arranged (“col/polyP-MP”). It is surprising that the size of the Ca–polyP nanoparticles that are formed onto the collagen scaffold is small (≈30–50 nm), compared to that of the Ca–polyP particles (450 ± 170 nm), formed in the absence of the collagen scaffold. At present we attribute the smaller size of the collagen-associated particles to an alteration of the surface tensions existing in the multi-phase hybrid system (collagen–polyP) versus the one phase (polyP) [42]. 

EDX experiments revealed that this hybrid material is indeed composed of polyP and collagen. With this latter completely physiological two-component biomaterial we intended to combine both morphogenetic activity elicited from polyP and the structural guidance of the collagen in the particles as well as the biological property to attract cells to attach and to support migration and differentiation in the vicinity of this fibrous meshwork. The structure guidance of the cells via integrin receptors (present on the cell surface) and the RGD domains within the collagen fibers allow directed migration of the cells and additionally also modulate morphogenetic signaling within the cells. These properties provide the key advantage of the collagen/Ca–polyP hybrid material in comparison to the pure amorphous Ca–polyP particle. In the in vitro studies performed here we showed that the amorphous microparticles “Mg–polyP-MP” and “Ca–polyP-MP”, but not the non-particulate Na–polyP (“Na–polyP”), significantly increase the expression of the collagen types *collagen-I* and *collagen-III*. A likewise significant upregulation of the steady-state-expressions of the two collagen genes is observed with the “col/polyP-MP” formulation. 

For the in vivo wound healing experiments both with normal and diabetic mice the three polyP preparations and also the original Na–polyP sample were tested. At two time points, after 7 days (Figure 6) and 13 days (Figure 7), the topical effects of the polymers were analyzed. Previously it has been found that during the 7-day healing period of acute wounds in normal mice the process is completed to about 50%, while after 13 days the healing is complete [43]. In diabetic mice the healing kinetics is delayed by two-fold. In the present animal study, we found that the re-epithelialization process in normal mice was completed by 30% after a period of 7 days, while only 22% epithelialization was measured in diabetic mice (see Figure 6 and Figure 7). In contrast to the wounds in normal mice, where an increase in the wound diameters is measured in response to a topical application of polyP, a significant reduction of the diameter is seen in diabetic mice using the same polymer samples. The increase in the wound size in normal mice could be attributed to a higher blood flow, perhaps caused by a local increase of NO, while the decrease in db/db mice could be indicative for an onset of fibroblast proliferation and differentiate into myofibroblasts [44]. In general, the topical treatment of wounds by polyP results in an increase of the wound healing. All polyP samples are significantly active. After 13 days the wound healing in normal mice is already complete, while in diabetic mice for “Ca–polyP-MP” and especially the host–guest “col/polyP-MP” groups, an increase of the re-epithelialization to 100% (complete cure) is measured. In contrast, the controls as well as the “Na–polyP” treated groups showed a similar wound healing state with around 30% re-epithelialization. This main part of the study here confers polyP, especially the “Ca–polyP-MP” and “col/polyP-MP” microparticles, a beneficial effect as an accelerator of wound healing especially in diabetic mice. This result provides a strong impact on future developments in topic wound healing therapy. In order to support these morphometric data by molecular biological experiments, qRT-PCR-based steady-state-expression determinations have been performed. As marker genes indicative for the wound healing progress the two collagen types (I and III) have been chosen; de-paraffinized tissue samples, taken from the wounds, have been applied. Those genes have been found to be upregulated in regenerating wound tissue [45]. The *collagen type I* gene and the additional marker gene *plasminogen activator inhibitor-1* (PAI-1; also termed endothelial plasminogen activator inhibitor, or serpin E1) were found to be significantly upregulated both in wild-type mice and db/db mice after a 7-day healing period. If the healing period was prolonged to 13 days, the transcript levels of all four genes were found to be upregulated if the wound area had been treated with “Ca–polyP-MPs” and “col/polyP-MPs” (host–guest particles). 

In conclusion, the presented findings support the conclusion that polyP, in the form of microparticles, causes beneficial anabolic effects on cells involved in wound regeneration. The question might be discussed as to why polyP in the form of microparticles, especially as Ca^2+^-salts, is superior to the highly soluble Na–polyP. As outlined above, that finding is on the basis of a delayed, beneficial release of the morphogenetically active Ca–polyP. Evidence has been presented that polyP is taken up by mammalian cells via endocytosis [8]. Since in the present study the size of the particles supplied to the cells is larger than the preferred particle size for the endocytic uptake machinery we postulate that only after ALP-mediated degradation of the microparticles are the nanoparticles generated taken up by the cells. In consideration of transfection studies it is established that the highly negatively charged DNA, like polyP, can be readily taken up by mammalian cells if entrapped into less charged particles, like liposomes [46]. Building on the presented in vitro and in vivo data we propose further safety studies in animals that might be followed by first human trials.
